# Morphology and Metal Binding Characteristics of a Natural Polymer—Kondagogu (*Cochlospermum gossypium*) Gum

**DOI:** 10.3390/molecules18078264

**Published:** 2013-07-15

**Authors:** V. T. P. Vinod, R. B. Sashidhar, Miroslav Černík

**Affiliations:** 1 Laboratory of Chemical Remediation Processes, Institute for Nanomaterials, Advanced Technology and Innovation (CXI), Technical University of Liberec, Studentská 1402/2, Liberec 1, 461 17, Czech Republic; 2 Department of Biochemistry, University College of Science, Osmania University, Hyderabad, Andhra Pradesh–500 007, India

**Keywords:** hydrocolloid, metal complexes, bioremediation, nanogel, kondagogu gum

## Abstract

Kondagogu (*Cochlospermum gossypium*) gum (KG), a natural tree exudate, was investigated for its morphological, adsorption and metal interaction behavior with various toxic heavy metals (Pb, Cd, Ni, Cr and Fe). SEM, AFM and TEM techniques were used to study the morphological changes occurring after metal adsorption onto the biopolymer structure. The degree of biosorption of metals on KG biopolymer surfaces was assessed by small-angle X-ray scattering analysis. EDXA spectrum revealed that the ion-exchange mechanism plays a major role in the binding process between KG and metal ions. The higher electron density observed in the KG-Cd complex suggests that Cd is strongly bound to KG compared to the other metals. This work provides a potential platform for developing a hydrocolloid-based nanogel for bioremediation of environmental contaminants.

## 1. Introduction

Gums are natural hydrocolloids and potential biopolymers for various food and nonfood applications. Carbohydrate polymers comprise more than 90% of the dry weight of all biomass. The term “gum” most often specifically denotes a group of industrially useful polysaccharides (glycans) or hydrocolloids and their derivatives that hydrate in hot or cold water to form viscous solutions or dispersions at low concentrations [1]. Gums are classified as being either natural or modified [2]. Natural gums include chitosan, seaweed extracts (e.g., alginates), plant exudates (e.g., *Arabic*, *tragacanth*, *karaya*, *kondagogu*), gums from seeds or roots (e.g., potato starch), and gums obtained by microbial fermentation (e.g., gum xanthan). Modified gums include mostly cellulose and starch derivatives, such as ethers and esters of cellulose. 

Natural plant derived gums, which are important natural forest products, are potentially valuable and useful as food additives, emulsifiers and pharmaceutical ingredients [3]. Extensive research work carried out by our group on kondagogu gum (KG), a tree gum sourced as a forest product by tribesmen, including its morphological, physico-chemical, structural, rheological, pharmaceutical and emulsifying properties, further established that this gum can also be used as a biosorbent for the removal of toxic metal contaminants from aqueous environments [4,5,6,7,8,9,10,11,12,13]. Structural analysis of this biopolymer has shown, it contains sugars such as arabinose, rhamnose, glucose, galactose, mannose, glucuronic acid and galacturonic acid. Based on spectroscopic characterization, the probable structural feature assigned to KG is (1→2)-β-d-Gal *p*, (1→6)-β-d-Gal *p*, (1→4)-β-d-Glc *p* A, 4-0-Me-α-d-Glc *p *A, (1→2)-α-l-Rha and (1→4)-α-d-Gal *p* A [6,7]. The major functional groups identified in the gum are hydroxyl, acetyl, carbonyl and carboxylic groups and the zeta potential of native gum was determined to be -23.4 mV, indicating that it contains negatively charged groups [6]. Recently, KG has been successfully employed in the bioremediation of toxic heavy metal ions such as Cd^2^^+^, Fe^2+^, Pb^2+^, Ni^2+^, and Cr^3+^. The biosorption percentage of Cd^2+^, Fe^2+^, Pb^2+^, Cr^3+^ and Ni^2+^ metal ions by KG was found to be 97.3 ± 1.4%, 86.3 ± 1.0%, 74.7 ± 0.8%, 52.4 ± 1.9% and 39.2 ± 0.5% (at pH 5.0 ± 0.1 and room temperature 25 ± 2 ^°^C), respectively [8,9,10,11].

Polymer metal complexes are used in various fields and their main applications are in organic synthesis as catalysts, environmental remediating agents, hydrometallurgy, sensing and biomedical fields [14]. Heavy metal sequestration of many natural biomaterials like chitosan, gellan and karaya gums and alginates have also been studied [15,16,17,18]. In these materials, the principal mechanisms of metallic cation sequestration involves the formation of complexes between a metal ion and functional groups (carbonyl, carboxyl, amino, amido, sulphonate, phosphate,* etc.*) present on the surface or inside the porous structure of the biosorbent. Different binding mechanisms such as ion exchange, physical adsorption, chemisorptions, complexation and micro-precipitation are proposed in metal binding processes by an adsorbent as well [19].

In the present study, KG, a natural polymeric hydrocolloid gel, form complexes with heavy metals. The morphologies of the metal complexes obtained and their metal binding characteristic were analyzed. Scanning Electron Microscopy (SEM), Atomic Force Microscopy (AFM) and Transmission electron Microscopy (TEM) techniques were used to visualize the native structures of a KG biopolymer and its metal complexes after metal adsorption. KG interacting with metal ions was assessed using Energy Dispersive X-ray Analysis (EDXA). Small-angle X-ray scattering (SAXS) techniques measures the particle sizes and the difference in electron density distribution mechanism of metals binding to KG.

## 2. Results and discussion

### 2.1. SEM-EDXA Analysis of KG and Its Metal Complexes

**Figure 1:**
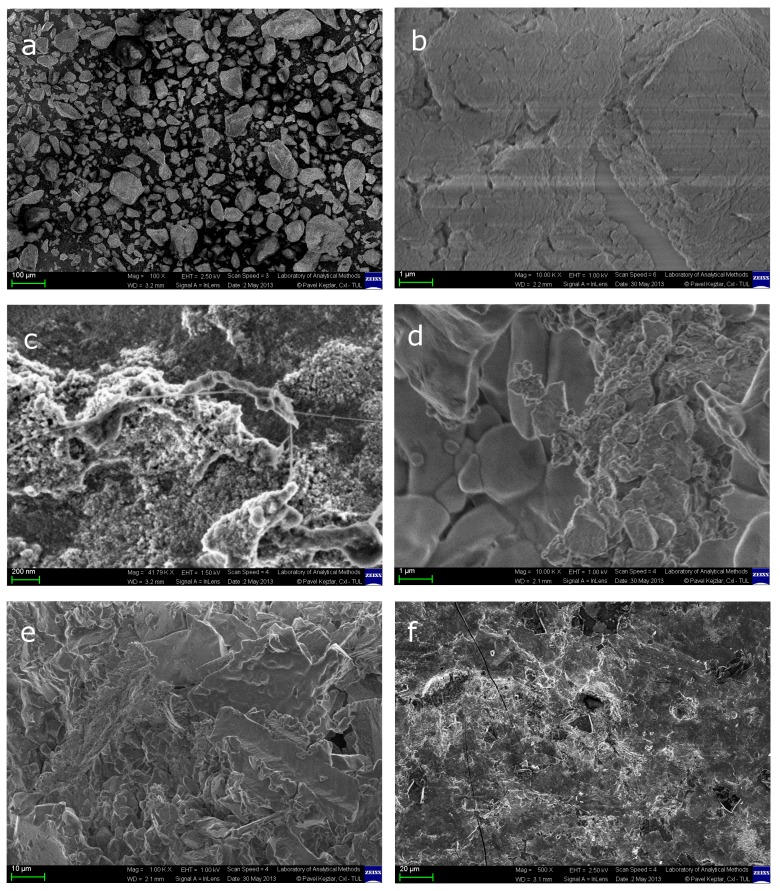
SEM micrographs of KG before and after metal biosorption, indicating morphological and structural changes (**a**) KG before metal biosorption; (**b**) KG-Pb complex; (**c**) KG-Fe complex; (**d**) KG-Ni complex; (**e**) KG-Cd complex; (**f**) KG-Cr complex.

Scanning electron microscopic examination of KG before and after metal biosorption (Pb^2+^, Cd^2+^, Ni^2+^, Cr^3+^, and Fe^2+^) was undertaken in order to locate the active adsorptive sites of the KG to form its metal complexes (Figure 1).

**Figure 2:**
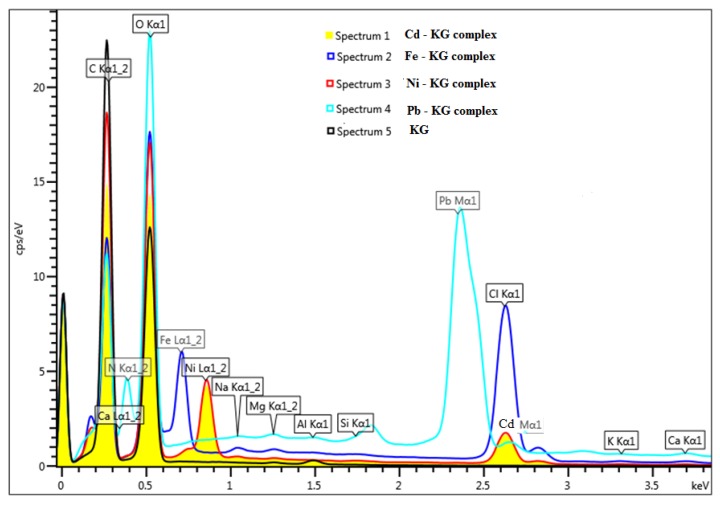
EDAX spectrum of KG before and after metal adsorption (Pb, Ni, Cd and Fe).

The energy dispersive X-ray analysis (EDXA) spectrum of KG before and after interaction with metal ions is shown in Figure 2. Scanning electron microscopy is a particularly useful tool for visual conformation of surface morphology and the physical state of the surface. SEM coupled with energy dispersive X-rays analysis (EDXA) was used to determine the metal uptake mechanism on KG, which could involve different adsorption mechanisms such as ion-exchange, complexation, and micro-precipitation. SEM images of the KG without metal Figure 1a binding show more irregular particle sizes with a porous structure on the surface. The EDXA spectrum of KG presented in Figure 2 shows that the major elements present are Na^+^, K^+^, Ca^2+^, Mg^2+^, Al, Si, Cl^−^, C, and O, respectively. The interaction between KG with Pb, Cd, Ni, Cr and Fe are depicted in Figure 1, suggesting the interaction of metal with hydroxyl, carboxyl, acetyl and carbonyl functional groups present in KG [6]. When KG was allowed to interact with a mixture of metal ions, metal cations replaced some of the alkali and alkaline earth metals (Na^+^, K^+^, Ca^2+^, Mg^2+^) naturally present in KG, suggesting an ion-exchange mechanism is the possible method of metal interaction between KG and added metal cations (Figure 2).

### 2.2. AFM Analysis of KG and Metal Complexes

**Figure 3:**
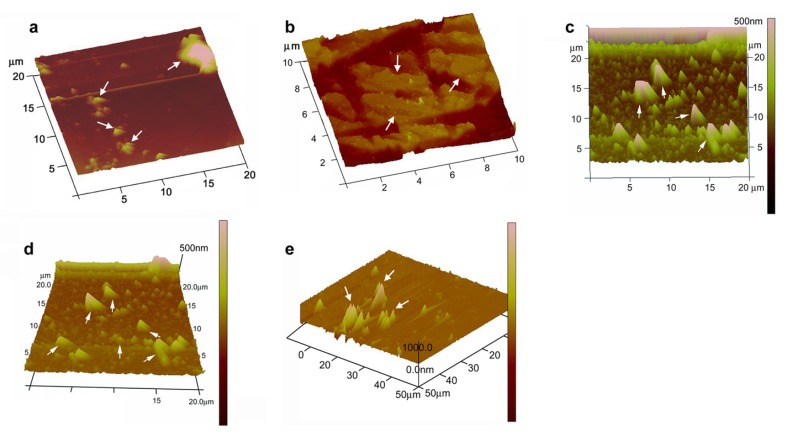
AFM images of KG-metal complexes indicating metal-capped structures of (**a**) KG-Pb; (**b**) KG-Cd; (**c**) KG-Ni; (**d**) KG-Fe and (**e**) KG-Cr.

Figure 3 shows the AFM image of KG-metal complexes. The results revealed that AFM can be used in the binding-structural studies of natural polymers and morphological changes of the sample can be attributed to the interactions between heavy metals and the surface of KG. Most polysaccharide solutions tend to form entangled networks when air-dried onto mica. The density of polymers within the network depends upon the initial polymer concentration but can vary across different areas of the mica. Some polysaccharides form gels when the polymer concentration exceeds a critical value [20]. This offers the possibility of forming and imaging thin aqueous polysaccharide gel networks by AFM. Earlier literature survey suggests that the AFM image of gum Arabic and soybean polysaccharide deposited from 1.0 mg L^−1^ aqueous solution was observed as spherical lumps, branched or linear chains or rod-like structures due to inter- and/or intra-molecular aggregation. A higher polymer concentration (10 mg L^−1^) resulted in large irregularly shaped structures due to the formation of solution droplets during air-drying of the sample solutions deposited on the mica [20].

A good number of polysaccharide solutions tend to form entangled networks when air-dried onto mica. Since, gum Arabic belongs to the arabino-galactan type of gums which mainly consists of negatively charged glucuronic acid, the AFM image of gum Arabic deposited onto a freshly cleaved mica surface from a 1.0 mg L^−1^ aqueous solution resulted in the formation and aggregation of polymer chains. However, a modified sample preparation was suggested by Ikeda* et al.*, 2005 [20] by adding 2 mM Tween-20 as a non-ionic surfactant along with the sample solution and depositing onto freshly cleaved mica and later air drying and imaging using AFM. This modified procedure was adopted in the present study for KG, which contains a high content of uronic acid [6,7].

### 2.3. TEM Analysis

**Figure 4:**
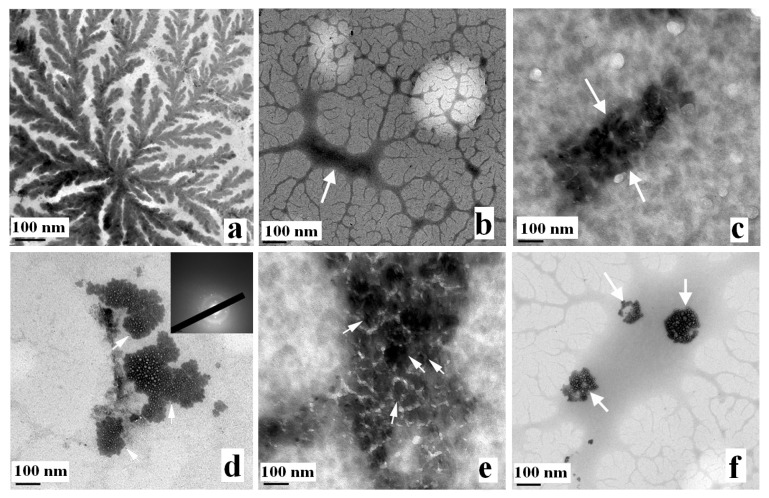
TEM images of KG and KG-metal complexes, indicating the formation of nanostructures. (**a**) pure KG (1.0 mg L^−1^); (**b**) KG-Cd complex; (**c**) KG-Pb complex; (**d**) KG-Ni complex; (**e**) KG-Fe complex; (**f**) KG-Cr complex.

TEM provides visual information on the wall thickness, size, shape and morphology of multilayer colloids. TEM images of the KG before and after metal interaction are presented in Figure 4. The concentration of KG (1.0 mg L^−1^) shows an elongated network of chain structures in Figure 4a. A concentration of 1.0 mg L^−1^ of KG was used for metal binding studies. It shows that no definable electron-dense layers exist in KG before metal adsorption, whereas the electron-dense layers and morphological changes appear after biosorption, verifying the metal adsorption on KG Figure 4b–f. Furthermore, differential morphology of the gum was also observed in relation to the type of metal sorbed. The TEM image after metal binding of Pb Figure 4c is in concurrence with earlier reported TEM analysis of Pb^2+^ adsorbed on cellulose/chitin beads [21].

### 2.4. Small Angle X-Ray Scattering Analysis

**Figure 5:**
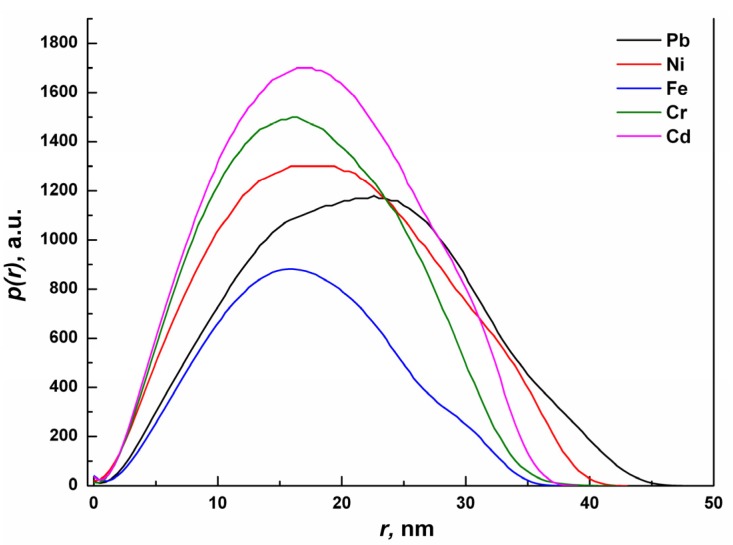
Plots of the experimental pair distance distribution function *p*(r)* versus* radial distance r of the KG-metal complexes, as determined by SAXS.

The pair distance distribution function p(r) obtained by SAXS analysis of KG-metal complexes is shown in Figure 5. The asymmetry in the p(r) curves reveals that the shapes of the particles are slightly deviated from the perfect spherical shape [22]. The curves exhibit distinct peaks for the unimodal distribution of metal complexes with mean particle size values ranges from 10 nm to 30 nm for Cd, Cr and Fe, whereas for Ni the distribution is wider up to 40 nm. In case of Pb, the mean particle size is shifter to a larger value. Since the SAXS results are considered to be much better statistical average in comparison to that of TEM results, the former exhibit the real size of the particles. 

**Figure 6:**
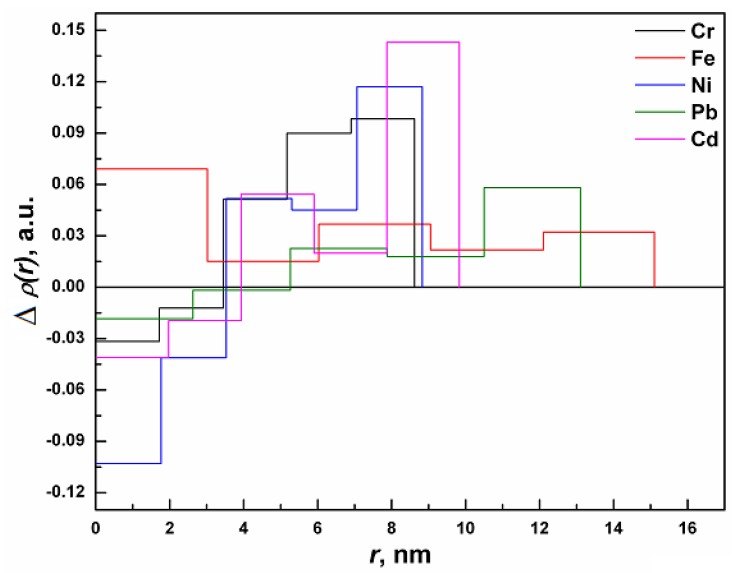
Resulting cross-section radial electron density profiles for GKG-metal complexes; Δp(*r*) calculated with the deconvolution procedure of p(*r*) using program DECON.

The electron density profiles generated using the program DECON [22,23] for the KG-metal complexes is shown in Figure 6. It can be seen that the electron density is not uniform, but it varies from the center to the surface for all of the samples (expect for Fe). In general, the electron density appears to be less at the surface due to the presence of a smaller number of atoms [24,25]. The observed higher electron density at the surface can be attributed to the capping of the gum matrix with metal ions. It is also observed that the obtained electron density profile was the highest for Cd^2+^ while it was the lowest for Fe^3+^. The above differences are probably attributed to the bonding/ interaction and capping ability of the individual metal ions with the various functional groups present in the KG matrix.

## 3. Experimental

### 3.1. Materials and Methods

KG samples were collected from Girijan Co-operative Corporation, Hyderabad, Government of Andhra Pradesh Undertaking, and M/S D.K. Enterprises, Hyderabad, India, who provided the samples free of charge. Gum kondagogu (Grade–1, hand-picked, fresh, and clean with no extraneous material), was used in the experimental analysis. The collected gum samples were stored in airtight polypropylene bottles in desiccated conditions. All other chemicals used were analytical reagent grade and used without further purifications.

### 3.2. Metal Solutions

All metal salts were of analytical grade materials and used for biosorption experiments without further purification. The salts of Pb(NO_3_)_2_, Cd(NO_3_)_2_ .4H_2_O, NiCl_2_·6H_2_O, FeCl_3_·6H_2_O and Cr(NO_3_)_2_ were used for making synthetic stock solutions. The oxidation states of the metals used were Cd^2+^, Fe^3+^, Pb^2+^, Ni^2+^, and Cr^3+^, respectively. The pH adjustments were made using either 0.1 M HCl or 0.1 M NaOH solutions. All the working solutions were prepared by diluting the stock solution in MilliQ water. 

### 3.3. Biosorbent Preparation

KG was powdered in a high-speed mechanical blender and later sieved using a bin (mesh size–250 μm), so as to obtain a fine and uniform sample. KG powder (1 g) was accurately weighed, and dispensed into a clean glass beaker containing one liter of deionized water. The whole gum solution was kept on a magnetic stirrer at room temperature and gently stirred overnight. Later, the gum solution was allowed to stand at room temperature (25 ^°^C) for 12 h to separate any suspended matter and foreign particles. The gum solution was filtered through a sintered glass funnel (#G-2 followed by #G-4). The clear solution obtained was freeze-dried and stored at room temperature until further use. The freeze-dried gum was used for the preparation of metal complexes.

### 3.4. Preparation of KG-Metal Complexes

KG-metal complexes were prepared by dispersing 100 mg of the lyophilized KG in 20 mL of 10 mM metal solutions (Cd^2+^, Fe^3+^, Pb^2+^, Ni^2+^, and Cr^3+^) and were shaken at 200 rpm (Innova 43, New Brunswick Scientific Co. Ltd, Edison, NJ, USA), for 120 min at room temperature. The contents were later centrifuged for 5 min at 10,000 × g and the supernatants were collected and subsequently filtered through a 0.45 μm micro filter. The metal ions were analyzed using inductively coupled plasma optical emission spectroscopy (Optima 2100 DV, Perkin Elmer, Waltham, MA, USA). The difference in equilibrium and initial metal concentration gives the amount of metal biosorbed by the gum. The heavy metal complexes and kinetics of metal biosorption by KG have been described in detail in our earlier communication [9,10,11].

### 3.5. SEM-EDXA Analysis

The surface structure of KG before and after biosorption was analyzed by scanning electron microscope coupled with energy dispersive X-ray analysis SEM-EDXA (ZEISS, Ultra/Plus, Jena, Germany, and EDS detector OXFORD Instruments X-Max 20, SW AZtec 2.1; Oxford, UK). The unloaded and metal-loaded KG samples were mounted on a stainless steel stab with double-sided adhesive tape with a thin layer of gold under high vacuum conditions. 

### 3.6. Atomic Force Microscopic (AFM) Analysis

The top surface morphology of the KG and KG-metal complexes were investigated with a Veeco Nanoscope IV controller (Digital Instruments, Santa Barbara, CA, USA). The instrument was operated in ambient air in the tapping (*i.e.*, intermittent contact) mode, using standard silicon probes with a resonant frequency of ~250 kHz.

### 3.7. Transmission Electron Microscopic (TEM) Analysis

Transmission electron microscopy was carried out on a transmission electron microscope (JEOL 100 CX, Tokyo, Japan). KG with and without metal complexes was fixed with a 2.5% (w/v) solution of glutaraldehyde-phosphate buffer (100 mM; pH 7.5) for 2 h, and later washed repeatedly with 100 mM phosphate buffer, dehydrated in a graded ethanol series (50%–100%) and acetone (100%). Resin infiltration was carried out overnight in 2:1 (v:v) acetone/resin, then further 4 h in 1:1 (twice) and finally overnight in 100% resin. The resin infiltration steps were performed at 20 °C and eventually the samples were transferred into molds filled with fresh spur resin for polymerization at 60 °C for 24 h [26].

### 3.8. Small Angle X-Ray Scattering (SAXS) Analysis

Small angle X-ray scattering measurements for the KG and KG-metal complexes were made with the help of a PW3830 X-ray generator (Anton–Paar GmbH, Graz, Austria) operated at 40 kV and 50 mA with a Cu target. The scattering data collected were used to calculate size, shape and distribution of the KG and KG-metal complexes. Data were obtained in the form of scattered X-ray intensity ‘I’ as a function of the scattering vector q = (4π/λ) sinθ [1/nm], where θ is the scattering angle and λ is the wavelength of the radiation. The data analysis was performed using the GIFT computer software program.

## 4. Conclusions

The experimental investigations illustrate the binding capacity of various metals with KG and the proposed mechanisms of binding to be complexation, micro-precipitation and ion-exchange. SEM, AFM and TEM analysis suggests that KG interacts with various metals (Cd, Cr, Ni, Pb and Fe) and forms metal-capped structures. The study shows that the KG is a highly effective polymeric gel to sequestrate toxic metals from an aqueous solution. The binding affinity of the toxic metal to KG descents in the order of Cd > Cr >Ni > Pb > Fe as determined by SAXS analysis. Based on the study the possibility arises to fabricate a nanogel based on KG that could be used for bioremediation of toxic heavy metals and other environmental contaminants from aqueous and industrial effluents. Furthermore, the present work could also be useful for the potential application of metal-polymer complexes as catalysts in chemical reactions, detection of pollutants in water, wastewater treatment and hydrometallurgy.
